# Stocktaking of the progress towards the implementation of the global manifesto on improving cancer care in conflict-impacted populations

**DOI:** 10.3389/fonc.2026.1826784

**Published:** 2026-06-10

**Authors:** Iman Ahmed, Samiratou Ouédraogo, Nazik Hammad

**Affiliations:** 1 African Organization for Research and Training in Cancer, Greater Toronto Area, Toronto, ON, Canada; 2 McGill University, Montreal, QC, Canada; 3 University of Toronto, Toronto, ON, Canada

**Keywords:** cancer, conflict, implementation, manifesto 2024, progress

## Introduction

**Figure 1:**
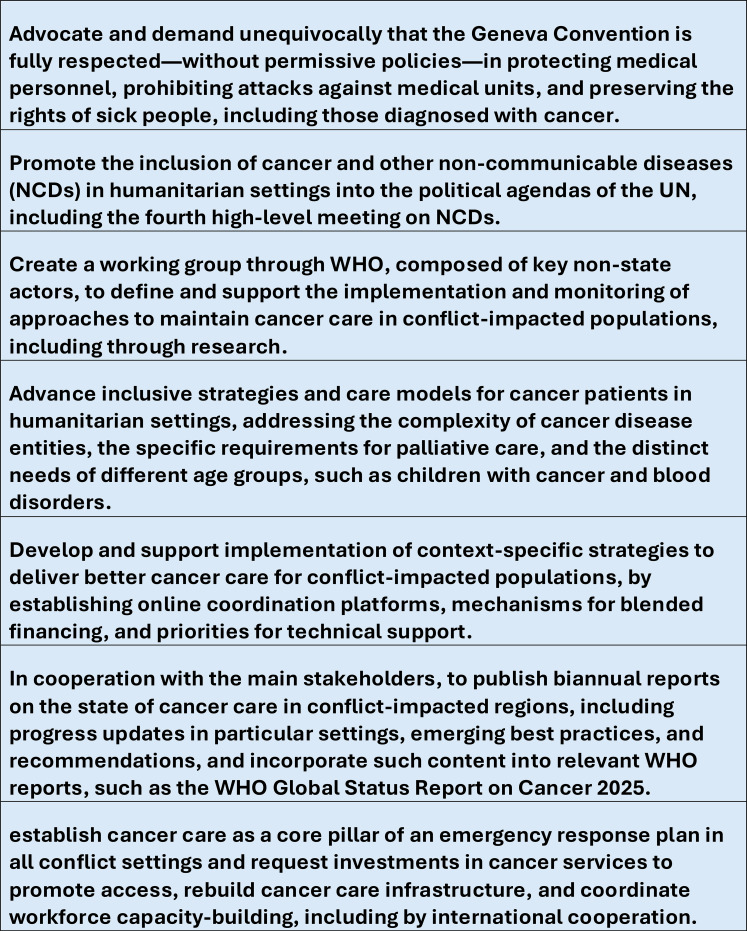
Seven recommendations of the WHO and partners manifesto on improving cancer care in conflict-impacted populations (Lancet 2024).

The manifesto on improving cancer care in conflict-impacted populations was published in August 2024, following the first Global Summit on War and Cancer, which took place on December 14–16, 2023 (1). The summit was organized by the Institute of Cancer and Crisis and OncoDaily, bringing together people (including renowned health-care experts, policy makers, and patient advocates) and organizations, all sharing a commitment to addressing the challenges in providing cancer care to conflict-affected populations in various parts of the world (2). The manifesto provided seven key recommendations (**Figure 1**). Unless a framework for implementing these recommendations is put in place, incorporating progress metrics and the challenges faced in monitoring progress, and ideally proposing ways to overcome these challenges, it is hard to envision how the manifesto could be moved from the point of recommendations to practical action coupled with measurable impact.

Despite the above reality, this paper attempts to establish baseline information on the progress made, or lack thereof, in implementing the recommendations of the manifesto and the stark disparities in cancer response in various humanitarian settings, which challenge the essence of global health equity and the principle of health for all (3, 4). The paper explores publicly available data and information available to the researchers through their participation in intersectional professional networks addressing cancer in conflict and displacement settings.

## Overview

A manifesto is by definition a public document, usually issued by a group (as compared to an individual) declaring, through ideas, opinions and views, the position of the group in relation to a specific subject, and sometimes laying out a plan of action for execution to address the status quo (5). A quick look at the global manifesto on improving cancer care in conflict-impacted populations demonstrates that it provides a set of seven recommendations yet stops short of putting forward a plan of action for the implementation of the recommendations provided. Thus, it is important to draw distinction between the manifesto as such and its implementation when taking stock of the progress.

The seven recommendations (**Figure 1**) can be summarized as advocating against attacks on healthcare, promoting the inclusion of NCDs (including cancer care) in humanitarian emergency response, creating -through the World Health Organization- a working group of Non-State Actors to support the cause and monitor actions, developing context-specific cancer care strategies, publishing a bi-annual report on the status of cancer care in conflict settings, and establishing cancer care as a core pillar of emergency response plans while requesting funding support – including through international cooperation - to advance cancer care for displaced populations across the health systems building blocks.

## Progress, regression, or a combination of both?

In this section, the paper will explore evidence, available through our research, on implementation of each of the seven recommendations of the manifesto. The level of evidence may vary, and thus our reporting will be commensurate with the data generated.

### Recommendation 1: “Advocate and demand unequivocally that the Geneva Convention is fully respected—without permissive policies—in protecting medical personnel, prohibiting attacks against medical units, and preserving the rights of sick people, including those diagnosed with cancer.”

Two years have passed since the commitments made at the Global Summit on War and Cancer. During this period, the world has been witnessing unprecedented threats to International Law. Between August 3rd2024 – the date of publication of the manifesto- and May 19th 2026 (the date of submission of this manuscript), the World Health Organization recorded a total of **1029** attacks on healthcare in 17 countries and territories, representing an ever escalating grave violation of the Geneva Conventions, collectively known as the “The Laws of War” (6, 7). These attacks have resulted in 1256 deaths and 1384 injuries, involving 639 incidents impacting health facilities, 387 incidents on ambulances and medical transport vehicles, 467 attacks on personnel and 128 on warehouses (6). Several national and international bodies, health and human rights organizations and advocacy groups have issued repeated calls for the protection of the health workforce, and some have proposed protection strategies during conflicts but most of these strategies are yet to be incorporated into national legislature. The phenomenon of attacks on healthcare has been increasing, with no clear end in sight and no signs of respect by warring parties on the global scale. Unless national and international mechanisms to end impunity and bring perpetrators to justice, the image looks grim on this front and the losses will continue to compound, affecting access to healthcare for cancer patients and the wider populations forced to live in conflict zones overall.

### Recommendation 2: “Promote the inclusion of cancer and other non-communicable diseases (NCDs) in humanitarian settings into the political agendas of the UN, including the fourth high-level meeting on NCDs.”

Significant progress has been made in this regard. WHO has played a major role in driving this change, building on its earlier leadership, successfully cementing the commitment of United Nations Member States in a landmark resolution (A/80/L.34) concerning the political declaration on the prevention and control of noncommunicable diseases (NCDs) and the promotion of mental health and well-being (8). The declaration was presented to Member States at the Fourth High-level Meeting on NCDs on 25 September 2025 and adopted on Monday 15th December 2025 (8). The resolution is considered a landmark political commitment, crowning years of steady global efforts from grassroots communities, healthcare workers, scientists, academics and researchers as well as policymakers and political leaders. Its value lies in the groundwork that preceded it, which continues to give it legitimacy, and guarantee progress.

While this is a very welcome progress, cancer, as a disease category, requires complex diagnostic, treatment approaches and continuity of care, and often complex therapeutic geographies with patients and families needing to seek care across borders and beyond (9). The cost of cancer care for refugees can be offset with investment in system readiness. A WHO (2020) report indicates that an investment of US$1.00 in cancer care for refugees yields US$2.30 in direct productivity and US$9.50 in full social return (10, 11). Recent research on internally displaced people in Nigeria, showed that cancer is the costliest NCD to patients and families in conflict (12).

From this point, the world can only move forward to ensure that the political commitment generated at the UN General Assembly is translated into actionable progress that is reflected in improvement of NCD services and cancer care for conflict-affected populations.

### Recommendation 3: “Create a working group through WHO, composed of key non-state actors, to define and support the implementation and monitoring of approaches to maintain cancer care in conflict-impacted populations, including through research.”

Multiple initiatives have been established by the WHO addressing specific types of cancer and focusing on childhood cancer (13). These include the global strategy to accelerate the elimination of cervical cancer as a public health problem, the global breast cancer initiative, and the CureAll global initiative for childhood cancer (14–16).

However, up to date, there is no evidence of the creation by WHO of a working group that focuses on cancer care in conflict and displacement settings. It is of utmost importance that WHO consolidates the various efforts by scholars, leading scientists, humanitarian and research organizations under one umbrella, while establishing the recommended working group and creating clear indicators for the reporting on the achievements by the working group.

### Recommendation 4: “Advance inclusive strategies and care models for cancer patients in humanitarian settings, addressing the complexity of cancer disease entities, the specific requirements for palliative care, and the distinct needs of different age groups, such as children with cancer and blood disorders.”

The integration of non-communicable diseases and mental health into primary healthcare has been advanced through country-level implementation of technical packages such as the WHO (PEN) package of essential non-communicable disease interventions or technical packages addressing key non-communicable diseases risk factors (17). However, access to adequate continuous treatment remains a challenge especially for patients in palliative care. Palliative care, survivorship, and long-term follow-up, are critical for holistic cancer care often neglected in low resource settings not to mention humanitarian ones. Despite their importance for quality of life and symptom management, they are often neglected undermining the assessment of full care delivery effectiveness in conflict-impacted populations.

As noted in the global vison for action from WHO and partners on integrating cancer into crisis, effective treatment of childhood cancers is associated with excellent survival but requires timely access, multidisciplinary approach and continuity of care (18). There have been programs to provide cross border care for children with cancer including medical evacuation. However, children in conflict affected zones in Africa remain without access to medical evacuation. Addressing the childhood cancer in war zones requires innovative and tailored transnational and regional approaches that enables prompt delivery of care with continuity of care.

### Recommendation 5: “Develop and support implementation of context-specific strategies to deliver better cancer care for conflict-impacted populations, by establishing online coordination platforms, mechanisms for blended financing, and priorities for technical support.”

In a recent publication titled “Cancer without borders: Policy frameworks for oncology care in humanitarian and conflict settings”, Parmer and Rathod provided a comparative analysis of the key challenges, response mechanisms, and policy gaps in cancer response in three protracted humanitarian emergency settings; Gaza, Sudan, and Ukraine (19). The demonstrated disparities in response mechanisms and policy gaps are stark, ranging from minimal response and total exclusion of cancer from emergency response and health cluster priorities in the case of Sudan, to EU-wide registry, fast-tracked cross-border care in the case of Ukraine.

Such disparities challenge the essence of global health and the principle of health equity. In the case of Sudan, while the limited funding of the humanitarian appeal may explain the strain on program funding, the lack of prioritization of cancer care by Ministry of Health, WHO Sudan and the Health Cluster are inexcusable.

Establishment of the recommended online coordination platforms, along with mechanisms for blended financing, and priorities for technical support may have contributed to leveling the stark disparities between continents when responding to cancer care in conflict settings. An online coordination platform would have provided more visibility in the case of Sudan and enhanced the opportunity for global collaboration among actors and opened the door for effective public private partnerships to change the status quo. In simple terms, we act on what we see, and what is invisible usually receives least attention. However, such platform still does not exist, nor do mechanisms for blended financing or a global coordinated initiative for the provision of technical support to cancer care in conflict and displacement settings.

### Recommendation 6: “In cooperation with the main stakeholders, to publish biannual reports on the state of cancer care in conflict-impacted regions, including progress updates in particular settings, emerging best practices, and recommendations, and incorporate such content into relevant WHO reports, such as the WHO Global Status Report on Cancer 2025.”

There is still time to achieve this recommendation, noting the date of issuance of the manifesto in 2024. Yet, there is an observable gap in leadership when it comes to compilation and publication, by WHO, of a report focusing on cancer care in conflict settings. Scholarly contributions continue to systematically document the evidence of the global burden of cancer among refugees (20). However, a global report with a dedicated focus requires a higher level of accountability and leadership from WHO and partners.

### Recommendation 7: “Establish cancer care as a core pillar of an emergency response plan in all conflict settings and request investments in cancer services to promote access, rebuild cancer care infrastructure, and coordinate workforce capacity-building, including by international cooperation.”

The majority of humanitarian settings especially in Sub-Saharan Africa are characterized by limited access to oncology services which is exacerbated by logistical barriers, political instability, and funding shortages, increasing morbidity and mortality among cancer patients (21, 22). Many programs rely heavily on external expertise and intermittent support, raising concerns about continuity and resilience during crises. Workforce shortages remain acute due to displacement, brain drain, and security concerns in most of these settings (23). Tailored innovative interventions such as priority setting, framework implementation, needs-based resource allocation, utilizing mobile clinics, telemedicine and patients’ navigation have proven effective in constrained conditions to maintain treatment continuity and mitigate adverse cancer impacts (24). Unfortunately, there is limited evidence on nation-wide implementation and sustainability of such interventions. Effective governance structures and coordinated multi-sector collaborations involving governments, NGOs, international organizations, public private partnerships, and local stakeholders are key to sustaining cancer care services during conflict. These models should enhance resource allocation, program implementation, and policy development tailored to fragile health systems (25, 26).

The lack of prioritization of cancer care in health emergency response packages – as demonstrated in the case of Sudan – shows that there is a long way to go in harmonizing the incorporation of cancer care in emergency response on a global scale (19).

## Discussion

Cancer caused approximately 10 million deaths in 2020, with 70% of these occurring in low- and middle-income countries but remains poorly represented in response to crises and emergency preparedness (27). Global conflict related displacement is on the rise, with 68.6 million out of the total 82.2 million IDPs at the end of 2025 being displaced as a result of conflict (28). Cancer care in conflict affected populations is now a critical global health care need.

The manifesto on improving cancer care in conflict-impacted populations was published nearly 2 years ago. It was followed by a policy review to Integrating cancer into crisis: a global vision for action from WHO and partners (18). Several proposed solutions are directly derived from the manifesto and seek to distill the recommendations into a roadmap and actionable solutions. These include integrating the cancer care continuum into national preparedness and response plans to enhance health-care system resilience; including cancer in humanitarian responses efforts; addressing the specific needs of pediatric patients with cancer; improving cancer intelligence and surveillance systems; and developing strategies to navigate the logistical and financial challenges of providing cancer care during crises.

Nearly two years after the publication of the manifesto and the follow up recommendations from the WHO and partners, the manifesto is still not very well known, and the global cancer community is yet to embrace and implement the recommendations and policy solution. Despite this, the last two years have seen a significant progress in terms on putting cancer in crisis settings on the agenda of global oncology and its platforms. However, the global attention to cancer in crisis has not been equitable in the sense that certain parts of the world in particular such as sub-Sarhan Africa have not received the same attention and international support whether in countries with conflicts that often house sizable internally displaced populations (IDPs) or in neighboring countries that host sizable refugee population (29).

Given the recent attention to cancer in conflict affected populations and the increasing prevalence, complexity and protractedness of conflicts, we would like to bring the world’s attention to the manifesto. The manifesto can serve as a policy framework to address the cancer care needs of conflict-affected populations whether in countries affected by conflict, countries hosting refugees and at regional and international levels. The manifesto can also inform conflict response and preparedness at the level of NCCP and in collaboration with all stakeholders, to produce actionable plans and metrics to improve the cancer intelligence and surveillance systems to address health economics and financing of cancer care in humanitarian setting. Research into the complex social, ethical, economic psychological and gender dimensions of cancer in conflict affected countries and zones should be funded and recognized as critical in the global oncology community in its various platforms including academic, education, and advocacy policy arenas.

## Conclusion

It is evident from this analysis exploring the progress towards implementing the recommendations of the manifesto on cancer care in conflict and displacement settings that an implementation framework with measurable indicators is highly needed. Creating a leadership team in the form of a working group would facilitate the monitoring of progress.

We hereby make a global call for the adoption and implementation of the WHO and partners manifesto on cancer in conflict affected population, creation of the necessary structures that include a working group to lead its implementation, establishing a consolidated online platform for information sharing and partnership creation including Public-Private-Partnerships (PPP), emphasizing the investment case in cancer inclusion into health emergency response and investing in workforce development to level the disparities and enable the achievement of better outcomes among cancer patients impacted by conflict and displacement across the globe.
